# Progress in the Total Synthesis of Rocaglamide

**DOI:** 10.5402/2011/239817

**Published:** 2011-04-04

**Authors:** Xiao-hua Cai, Bing Xie, Hui Guo

**Affiliations:** ^1^College of Chemistry and Environmental Science, Guizhou University for Nationalites, Guiyang 550025, China; ^2^College of Pharmaceutical Sciences, Zhejiang University of Technology, Hangzhou 310014, China

## Abstract

The first cyclopenta[b]benzofuran derivative, rocaglamide, from *Aglaia elliptifolia*, was found to exhibit considerable insecticidal activities and excellent potential as a therapeutic agent candidate in cancer chemotherapy; the genus *Aglaia* has been subjected to further investigation. Both the structural complexity of rocaglamide and its significant activity make it an attractive synthetic target. Stereoselective synthesis of the dense substitution pattern of these targets is a formidable synthetic challenge: the molecules bear five contiguous stereocenters and cis aryl groups on adjacent carbons. In past years of effort, only a handful of completed total syntheses have been reported, evidence of the difficulties associated with the synthesis of rocaglate natural products. The advance on total synthesis of rocaglamide was mainly reviewed from intramolecular cyclization and biomimetic cycloaddition approach.

## 1. Introduction

**Scheme 1:**
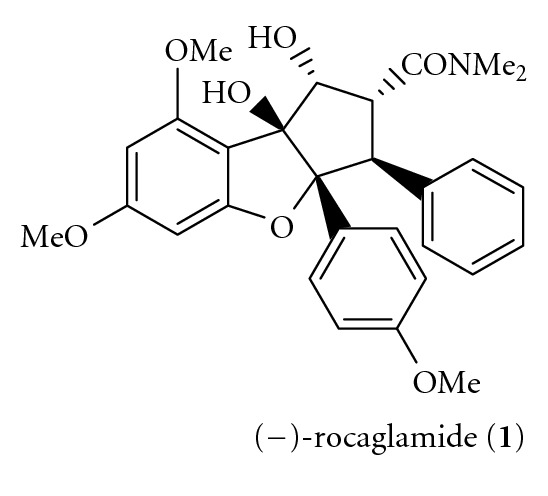


During the past few years, indigenous to southeast Asia, the plant genus *Aglia* includes several species that produce a range of cyclopenta[b]tetrahydrobenzofuran containing metabolites [1–3], including rocaglamide **(1)**, isolated from the roots and stems of *Aglia elliptifolia* by King et al. [4]. King's initial report indicated that rocaglamide showed significant in vivo activity in P388 lymphocytic leukemia-infected mice [4]. Since then, rocaglamide and related compounds have shown cytostatic, and cytotoxic activity against a variety of human cancer cell lines, with IC_50_ values in the range 1.0–6.0 ng/mL [5–9], has attracted more attention in recent years because of its insecticidal and growth inhibitory activity [10–15]. In order to be useful as drugs, a constant supply of such compounds in a large quantity is required. However, their natural abundance in the plant is quite low, and large-scale isolation from natural sources may not be feasible. Chemical synthesis, either total- or semisynthesis, is an option to produce this type of compounds. Both the structural complexity of rocaglamide and its significant activity make it an attractive synthetic target. Stereoselective synthesis of the dense substitution pattern of these targets is a formidable synthetic challenge: the molecules bear five contiguous stereocenters and cis aryl groups on adjacent carbons. In past years of effort, only a handful of completed total syntheses have been reported, evidence of the difficulties associated with the synthesis of rocaglate natural products. In the present work, several total synthetic approaches of rocaglamide will be reviewed (Scheme 1).

## 2. Intramolecular Cyclization Approaches

### 2.1. Synthesis of Di-Epi-Rocaglamide

**Scheme 2:**
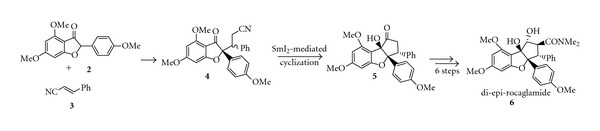
Synthesis of di-epi-rocaglamide.

An earlier attempt to synthesize rocaglamide **(1)** by Kraus and Sy in 1989 resulted in the synthesis of the di-epi analog of rocaglamide **(6)**, as shown in Scheme 2 [16]. Michael addition of benzofuranone **2** to cinnamonitrile **3** gave keto-nitrile **4** in a 5 : 1 diastereomeric ratio. The major isomer was used to prepare **5** via an SmI_2_-mediated cyclization, followed by the introduction of the dimethylcarboxamido group in six steps to give **6**. Although the investigators did not succeed in synthesizing rocaglamide **(1)**, this approach was the first to utilize pinacolic coupling to generate the cyclopenta[b]benzofuran skeleton. Intramolecular pinacolic coupling later became a routine methodology used by other groups in the synthesis of rocaglamide and rocaglate derivatives [17–20]. 2,3-Di-epi-rocaglamide **(6)** is an interesting compound that can be used in the SAR study of rocaglamide derivatives.

### 2.2. Synthesis of Racemic (±)-Rocaglamide

**Scheme 3:**
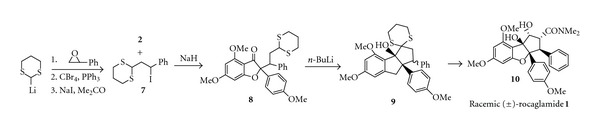
Synthetic approach (a) of racemic (±)-rocaglamide.

Davey and Taylor [21] were the first research group to utilize benzofuranone** 2** as the precursor in the synthesis of cyclopenta[b]benzofuran skeleton. Treatment of benzofuranone **2** with NaH followed by iododithiane **7** gave the C-alkylated product **8**, which, through a direct 1,3-dithiane lithiation and an intramolecular carbonyl addition gave the cyclized product **9 **as shown in Scheme 3. In order to complete the synthesis of rocaglamide** (1)**, all that remained were dithiane hydrolysis, introduction of the C-2 dimethylcarboxamide group, and carbonyl reduction. However, as reported in a follow-up publication, hydrolysis of dithiane **9** failed to give rise to the required *β*-phenyl isomer, which has the right stereochemistry for rocaglamide type compounds [18]. Under different reaction conditions, only the *α*-phenyl isomer was obtained in very low yield, and attempts to invert the stereochemistry also failed [18].

**Scheme 4:**
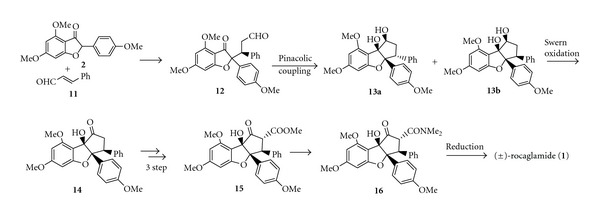
Synthetic approach (b) of racemic (±)-rocaglamide.

Taylor et al. [17, 18] then followed an alternative synthetic strategy utilizing the intramolecular keto-aldehyde pinacolic coupling as outlined in Scheme 4. Michael addition of the benzofuranone **2** to cinnamaldehyde **(11)**, followed by SmI_2_-mediated intramolecular pinacolic coupling of ketoaldehyde **12**, gave diols **13a **and** 13b**, which could be separated by chromatography. Swern oxidation of diol **13b** yielded ketone** 14**, which was converted into *β*-keto ester **15** using the CS_2_-based procedure as utilized by Kraus and Sy [16]. The keto ester** 15 **was then converted into ketoamide **16**, followed by a stereoselective reduction with Me_4_NBH(OAc)_3_ to give (±)-rocaglamide **(1)**.

**Scheme 5:**
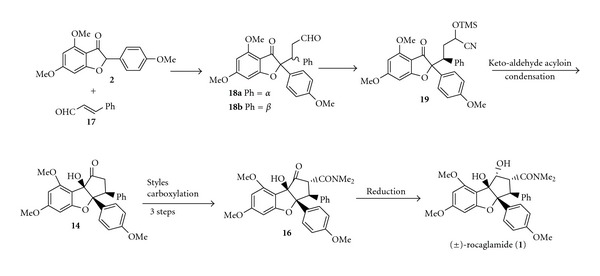
Synthetic approach (c) racemic (±)-rocaglamide.

In 2001, Dobler et al. modified Taylor's method [18, 20] to give a higher overall yield of rocaglamide (40%) in a fewer number of steps, as outlined in Scheme 5 [22]. Following Taylor's scheme, aldehyde was synthesized in 57% yield [18, 20]. Dobler et al. then proceeded with an umpolung sequence, where aldehyde **18b **was subjected to treatment with TMSCN to give the cyanohydrin **19 **in a quantitative yield, followed by a deprotection to give ketone **14 **[22]. Compared to Taylor's scheme, Dobler's method is compatible with substituents sensitive to reduction. For the introduction of the dimethylcarboxamide group, Dobler et al. utilized Styles reagent to convert the ketone **14** directly to ketomide **16 **[22]. In the final reduction step, both the groups of Taylor and Dobler used Me_4_NBH(OAc)_3_ to give an 81% yield (Taylor) [18] and a 95% yield (Dobler) [22] of racemic (±)-rocaglamide** (1)** (the yield is calculated from ketomide **16**).

**Scheme 6:**
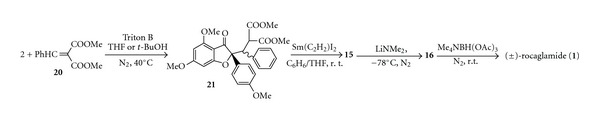
Synthetic approach (d) racemic (±)-rocaglamide.

In 2008, Qin's group synthesized (±)-rocaglamide and its 2,3-di-epi analogue by introducing the strategy of intramolecular reductive coupling to construct the cyclopenta[b]benzofuran skeleton [23], the shortest and most efficient synthetic method hitherto was now established to rocaglamide** 1 **and its 2,3-di-epi analogue in racemic form, as outlined in Scheme 6. Michael addition of the benzofuranone (**2**) to methyl *α*-formylcinnaminate **(20)**, followed by SmI_2_-mediated intramolecular keto-ester coupling, gave *β*-keto ester **15**, amination of the ester intermediate, and reduction of carbonyl with Me_4_NBH(OAc)_3_ to give (±)-rocaglamide **(1)**. 

**Scheme 7:**
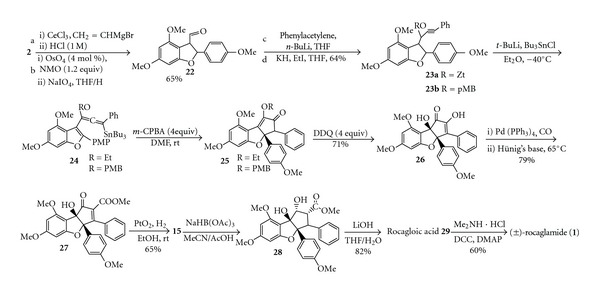
Synthetic approach (e) racemic (±)-rocaglamide.

 In 2009, Frontier group reported the total synthesis of aglafolin, rocagloic acid, and rocaglamide using Nazarov cyclization initiated by peracid oxidation (Scheme 7) [24]. Alkylation of benzofuranone (**2**) using vinyl magnesium bromide was followed by osmylation and periodate cleavage of the resulting 3-vinyl benzofuran to give aldehyde **22**. Alkylation with phenylacetylene and protection of the resultant propargyl alcohol with ethyl iodide and *p*-methoxybenzyl chloride gave propargyl ethers **23a **and **23b**, respectively. Deprotonation at the propargylic position of **23** with tertbutyllithium gave rise to an allenyl anion, which was trapped with tri-*n*-butyltin chloride to give stannyl alkoxyallene **24 **[25]. Treatment of **24 **with excess m-CPBA gave **25**, and treatment of **25b** with excess DDQ gave diosphenol **26** in excellent yield. Enol** 26** was converted to triflate and then subjected to palladium-mediated carbonylation to install the final C-C linkage and produced **27. **Hydrogenation of **27** over PtO_2_ gave **15** as a single diastereomer. Templated reduction of the ketone afforded the natural product aglafolin and saponification followed by amide formation furnished (±)-rocaglamide **(1)**.

## 3. Biomimetic Cycloaddition Approaches

**Scheme 8:**
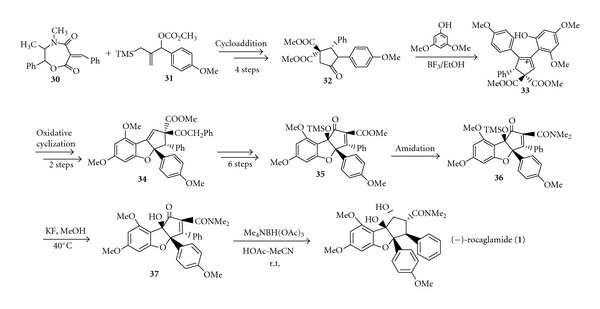
Synthesis of (−)-rocaglamide.

Trost et al. were successful in the enantioselective synthesis of (−)-rocaglamide (**1**), by utilizing a novel DDQ- (2,3-dichloro-5,6-dicyano-1,4-benzoquinone) mediated oxidative cyclization to generate the dihydrobenzofuran ring [26]. Some important steps for the formation of key intermediates are shown in Scheme 8. Trost et al. employed a Pd-catalyzed asymmetric [3+2] cycloaddition of TMM [(trimethylsilyl) methyl] precursor (**30**) and oxazepinedione (**31**) to give cyclopentanone (**32**), followed by condensation with dimethylphloroglucinol to give the adduct (**33**). A DDQ-mediated oxidative cyclization gave the dihydrobenzofuran ring (**34**), and the adjustment of the stereochemistry proceeded through the enone **35**. Amidation and desylation of (**35**) gave (**36**) and **37**, and reduction of **37** yielded (−)-rocaglamide** (1)**, which was identical to the natural product as shown by its chromatographic, spectroscopic, and physical properties. This synthetic method gave the enantiomerically pure rocaglamide and consisted of 17 steps with <6% overall yield [26]. 

**Scheme 9:**
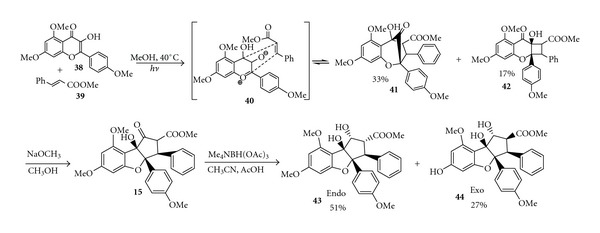
Synthesis of (±)-methyl rocaglate.

In 2004, Gerard group introduced a biomimetic approach to the rocaglates employing photogeneration of oxidopyryliums derived from 3-hydroxyflavones (Scheme 9) [27]. [3+2] cycloaddition of photoirradiation (uranium filter) of kaempferol derivative 3-hydroxyflavone **38 **and methyl cinnamate **39** (MeOH, °C) afforded the aglain **41**, as well as benzo[b]cyclobutapyran-8-one **42** (33% and 17%, resp.) after purification on SiO_2_. Basic conditions (NaOMe, MeOH) were used to perform *α*-ketol rearrangement of both** 41 **and **42** which afforded a mixture of endo and exo cycloadducts **15** in which the endo isomer was obtained as a mixture of keto-enol tautomers. Reduction of **15** afforded (±)-methyl rocaglate **43** (51%) and the corresponding exo stereoisomer **44** (27%).

**Scheme 10:**
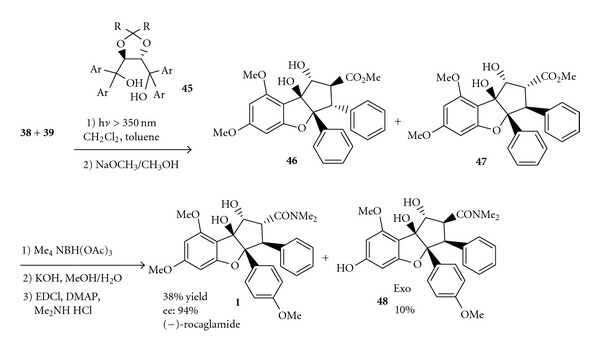
Enantioselectivity synthesis of (−)-rocaglamide.

Subsequently, Gerard group completed the asymmetric synthesis of the rocaglamides by enantioselective photocycloaddition mediated by chiral Brønsted acids [28] (Scheme 10). The approach involves enantioselective [3+2] photocyclo-addition promoted by chiral Brønsted acids (TADDOLs) to afford an aglain precursor followed by a ketol shift/reduction sequence to the rocaglate core, and the highest enantioselectivity of (−)-rocaglamide was 89% ee.

## 4. Other Synthetic Approaches

**Scheme 11:**
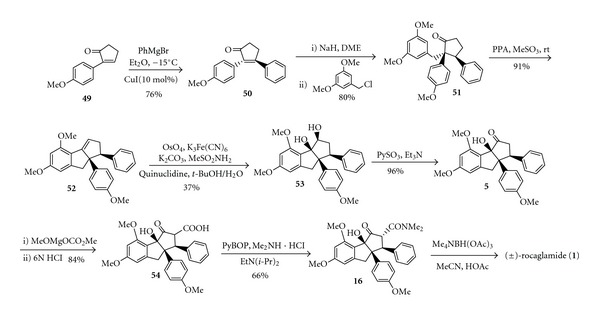
Synthesis of analogue of (±)-rocaglamide** (1)**.

**Scheme 12:**
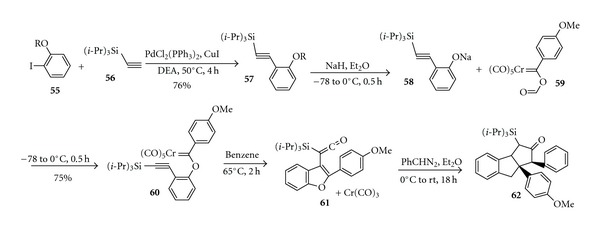
Stereoselective synthesis of the rocaglamide skeleton.

Bruce et al. synthesized the analogue of (±)-rocaglamide** (1)** by ten steps reactions from cyclopentanone, as shown in Scheme 11 [29]. A key feature of this route is a highly efficient intramolecular condensation reaction which cleanly leads to the tricyclic skeleton. In 2008, Giese and Moser [30] carried out stereoselective synthesis of the rocaglamide skeleton via a silyl vinylketene formation [4+1] annulation sequence (Scheme 12), and this novel approach affords the ABC ring system where the adjacent phenyl and aryl substituents of the C ring have the required cis relationship.

To summarize, in past years of effort, the synthetic methods of the rocaglamide have been developed rapidly, but valuable approaches are still few. At present, only intramolecular cyclization and biomimetic cycloaddition are effective and applied approaches for the synthesis of rocaglamide. It is very essential to perfect asymmetric Michael cycloaddition by the rigidity of molecular in intramolecular cyclization approach and to increase the synthetic total yield and region and stereoselectivity of the cycloaddition reaction in biomimetic cycloaddition approach. In the future, we believe more novel and effective approaches for the synthesis of rocaglamide will be developed.
